# A study on the correlation between APOE gene polymorphism, white matter hyperintensities, and neuropsychiatric symptom phenotypes in Alzheimer’s disease

**DOI:** 10.3389/fpsyt.2026.1795598

**Published:** 2026-03-20

**Authors:** Wei Fan, Ziqi Wang, Shu Wan, Man Liu, Yuanyuan Han, Xiaowei Liu, Lisi Xu, Xiaoyan Wang, Dingyi Zhang, Qingyan Cai

**Affiliations:** The Fourth People’s Hospital of Chengdu, Chengdu, China

**Keywords:** Alzheimer’s disease, Apolipoprotein E genotypes, mediation model, neuropsychiatric symptoms, white matter hyperintensities

## Abstract

**Objective:**

This study investigates the independent and interactive effects of apolipoprotein E (APOE) genotypes and white matter hyperintensities (WMH) on distinct neuropsychiatric symptom (NPS) phenotypes in patients with Alzheimer’s disease (AD).

**Methods:**

We enrolled 325 AD patients consecutively diagnosed at a specialized memory clinic between May 2024 and May 2025. All participants underwent comprehensive clinical assessments—including the Chinese Mini-Mental State Examination (CMMSE), Activities of Daily Living (ADL) scale, and the Neuropsychiatric Inventory (NPI)—as well as 3T brain MRI for WMH quantification and APOE genotyping. First, we compared NPS profiles and cognitive/functional scores across APOE genotype groups (ϵ2/ϵ2–ϵ2/ϵ3, ϵ3/ϵ3, ϵ3/ϵ4, ϵ4/ϵ4) using analysis of variance (ANOVA) or Kruskal–Wallis tests, as appropriate. Second, we applied mediation analysis (PROCESS macro Model 4, 5,000 bootstrap samples) to examine whether WMH burden mediates the association between APOE genotype (X) and outcomes including CMMSE total score and domain-specific NPS subscores (delusions, agitation, irritability, euphoria).

**Results:**

Significant differences emerged across APOE genotypes in both cognition (CMMSE, p < 0.05) and functional status (ADL, p < 0.05). At the symptom level, carriers of at least one ϵ4 allele exhibited higher agitation scores than non-carriers (p < 0.05); notably, the ϵ4/ϵ4 homozygotes showed significantly greater severity in delusions, agitation, irritability, and euphoria compared with all other genotype groups (all p < 0.05). Mediation analyses revealed no statistically significant indirect effect of APOE genotype on any outcome via WMH, indicating that WMH does not mediate these associations. Instead, APOE genotype exerted robust direct effects on both cognitive performance and specific NPS domains.

**Conclusion:**

APOE genotype—particularly the ϵ4/ϵ4 homozygous status—is associated with more pronounced cognitive decline and a distinct, severe NPS profile in AD, especially involving delusions, agitation, Euphoria, and irritability. These associations are independent of WMH burden, suggesting that APOE exerts direct neurobiological effects on neuropsychiatric manifestations. Thus, APOE genotyping holds dual clinical value: not only as a well-established biomarker for AD risk and diagnosis but also as a potential prognostic indicator for behavioral and psychological symptoms—offering actionable insights beyond conventional neuroimaging markers.

## Introduction

Alzheimer’s disease (AD) is the most common cause of dementia and a rapidly escalating global health priority, imposing profound personal, familial, and socioeconomic burdens. It ranks among the costliest, deadliest, and most disabling conditions of the 21st century (1). Although historically defined by its cognitive deficits, AD is now recognized as a multifaceted neurodegenerative disorder in which neuropsychiatric symptoms (NPS) are not merely comorbid but nearly universal—occurring in over 90% of patients across disease stages (2). NPS comprise 12 well-validated phenotypes, including hallucinations, delusions, agitation, anxiety, depression, and motor disturbances (3). These manifestations significantly erode patients’ quality of life, intensify caregiver distress and burnout, and elevate risks to patient safety and public health.

NPS in AD are not merely epiphenomena of the core neuropathological cascade; rather, they reflect complex interactions between neurodegenerative processes and modifiable biological factors. Emerging evidence implicates apolipoprotein E (APOE) genotype and white matter hyperintensities (WMH) as key contributors to NPS heterogeneity and severity in AD (4, 5). Human APOE has three major isoforms—ApoEϵ2, ApoEϵ3, and ApoEϵ4—each encoded by distinct allelic combinations: ϵ2/ϵ2 and ϵ2/ϵ3 for ApoEϵ2; ϵ2/ϵ3 and ϵ3/ϵ3 for ApoEϵ3; and ϵ3/ϵ4 and ϵ4/ϵ4 for ApoEϵ4. Within the central nervous system, ApoE supports essential physiological functions, including synaptic plasticity, lipid homeostasis, neuroinflammation modulation, neuronal repair, and memory consolidation. Critically, the APOE ϵ4 allele remains the strongest established genetic risk factor for late-onset AD, with dose-dependent effects on disease onset, progression, and neuropathological burden (6, 7). Beyond its well-established role in modulating AD risk, the APOE ϵ4 allele is increasingly recognized as a robust genetic modifier of NPS expression in established AD—contributing to interindividual heterogeneity in symptom profiles, severity, and trajectory (8). Empirical studies consistently report that AD patients carrying at least one ϵ4 allele exhibit significantly higher prevalence and severity of psychotic symptoms (e.g., hallucinations, delusions), affective disturbances (e.g., depression, anxiety), and apathy compared with ϵ4-noncarriers (9, 11, 12). A large-scale longitudinal study (n = 3,932) further demonstrated that APOE genotype significantly modifies the association between baseline apathy severity and subsequent dementia conversion, independent of cognitive status (10). Quantitatively, meta-analytic evidence indicates that ϵ4 carriers with AD face a 19-fold increased odds of hallucinations (OR = 19.0, 95% CI: 7.2–50.1) and a 3.4-fold elevated odds of delusions (OR = 3.4, 95% CI: 2.1–5.5) relative to non-carriers (13). These findings underscore APOE ϵ4 not only as a susceptibility factor for AD onset but also as a clinically relevant biomarker for NPS vulnerability. Despite accumulating evidence, the relationship between APOE genotype and NPS in AD remains incompletely characterized, with existing studies reporting inconsistent associations—some detecting robust genotype–symptom links, others finding no significant effects (4). WMH, a neuroimaging marker of cerebral small vessel disease, were long regarded as an incidental finding of normative brain aging. Contemporary evidence now firmly establishes WMH as an independent, dose-dependent risk factor not only for cognitive decline and dementia incidence but also for the onset and exacerbation of diverse neuropsychiatric manifestations—including depression, apathy, agitation, and executive dysfunction (14, 15). Critically, emerging neuropathological and neuroimaging data indicate that the APOE ϵ4 allele is associated with accelerated small vessel pathology, particularly in frontal and temporal periventricular regions. Mechanistically, ϵ4 may promote blood–brain barrier disruption, impaired vascular clearance, and chronic hypoperfusion—leading to increased WMH accumulation, which in turn contributes to network disconnection, synaptic dysfunction, and ultimately, the expression of NPS in AD (16).

Why do individuals with AD exhibit marked heterogeneity in NPS expression—and why do some patients develop severe, treatment-refractory symptoms while others remain relatively spared? This clinical variability reflects the interplay of genetic susceptibility, cerebrovascular pathology, and neural circuit vulnerability, underscoring an urgent need to delineate precise biological mechanisms. Building on prior evidence linking APOE genotype and WMH to NPS, this study systematically examines their independent and interactive contributions to NPS phenotypic diversity in a well-characterized AD cohort. We hypothesize that APOE ϵ4 homozygosity (ϵ4/ϵ4) is associated with a distinct, high-burden NPS profile—particularly involving psychosis, agitation, and apathy—beyond its known effects on amyloid burden and neurodegeneration. Critically, we test whether WMH mediates this association: specifically, whether APOE ϵ4 drives regionally selective small vessel injury (e.g., in frontal-striatal and limbic circuits), thereby disrupting structural and functional connectivity and ultimately facilitating the emergence of specific NPS domains. To this end, we integrate multivariate correlation analyses with formal mediation modeling (PROCESS Model 4) to quantify direct and indirect pathways from APOE genotype → WMH burden → NPS severity across six validated symptom clusters. By identifying genetically anchored, imaging-quantifiable pathways underlying NPS heterogeneity, our findings aim to advance precision psychiatry in AD—enabling earlier risk stratification, biologically informed intervention targets, and family-centered strategies to alleviate the profound clinical, functional, and psychosocial consequences of these symptoms.

## Methods

The study included 325 patients diagnosed with Alzheimer’s disease who attended the Memory Clinic at Chengdu Fourth People’s Hospital between May 2024 and May 2025. Inclusion criteria: (1) Fulfillment of the 2011 NIA-AA criteria for probable Alzheimer’s disease (AD); (2) Clinical diagnosis of probable AD confirmed by at least two psychiatrists with associate chief physician qualifications or higher; (3) Completion of comprehensive cognitive and functional assessments, including the Chinese Mini-Mental State Examination (CMMSE), Activities of Daily Living scale (ADL), and Neuropsychiatric Inventory (NPI), along with APOE genotyping, and acquisition of Siemens 3T magnetic resonance imaging (MRI) within one week of clinical evaluation at the Memory Clinic; (4) Availability of a reliable caregiver to provide consistent collateral history and support throughout participation; (5) Formal approval of the study protocol by the Institutional Ethics Committee of our hospital; (6) Provision of written informed consent by the patient or their legally authorized representative after full explanation of the study objectives, procedures, and potential risks.

The exclusion criteria were defined as follows: (1) Presence of other types of dementia such as vascular dementia(VaD: with a clear obvious dementia syndrome occurring within six months after cerebrovascular events), frontotemporal dementia(FTD),Lewy body dementia(DLB), etc., or presence of diseases that may cause other dementias or cognitive impairments such as head trauma, brain tumors, epilepsy, cerebral inflammatory diseases, demyelinating diseases of central nervous system, normopressure hydrocephalus, severe liver or kidney dysfunction, severe anemia, hypothyroidism, vitamin B12 deficiency, and so on. (2) Presence of white matter hyperintensities or other types of white matter lesions resulting from radiation injury, carbon monoxide poisoning, multiple sclerosis (MS), vasculitis, or cerebral white matter malnutrition.(3) Clear history of cerebrovascular disease with a Hachinski Ischemic Score (HIS)>4 and Fazekas score≥2.(4) Clear history of psychiatric disorders such as schizophrenia or severe depression (HAMD scale score<8), or clear history of substance abuse including drugs and alcohol.(5) Accompanied by severe disorders, severe aphasia, or inability to complete neuropsychological tests.

### Data acquisition

Demographic data, including age, gender, occupation, and handedness, were collected. Additionally, a comprehensive assessment of past medical history, smoking history, and alcohol consumption was conducted.

Cognitive assessment was performed by trained professional evaluators in the memory clinic who had undergone standardized training to ensure inter-rater reliability. The Mini-Mental State Examination (CMMSE) was administered, with a maximum total score of 30. The CMMSE comprises ten items across six domains: orientation (10 points), registration and immediate recall (3 points), attention and calculation (5 points), delayed recall (3 points), language (8 points), and visuospatial ability (1 point). Functional capacity was assessed using the Activities of Daily Living Scale (ADL), which yields a total score of up to 80. It consists of two subscales: the Basic Activities of Daily Living (BADL) with 11 items and the Instrumental Activities of Daily Living (IADL) with 9 items. Higher scores indicate greater functional impairment. Neuropsychiatric symptoms were evaluated using the Neuropsychiatric Inventory (NPI), administered through structured interviews with reliable caregivers. The NPI assesses 12 domains: delusions, hallucinations, agitation/aggression, depression/dysphoria, anxiety, euphoria/elevated mood, apathy/indifference, disinhibition, irritability/lability, motor disturbance, night-time behaviors, and appetite and eating changes. Each domain is scored based on frequency, severity, and caregiver distress, yielding a total possible score of 144. Higher total scores reflect more severe neuropsychiatric symptom burden.

### MRI data acquisition

**Figure 1:**
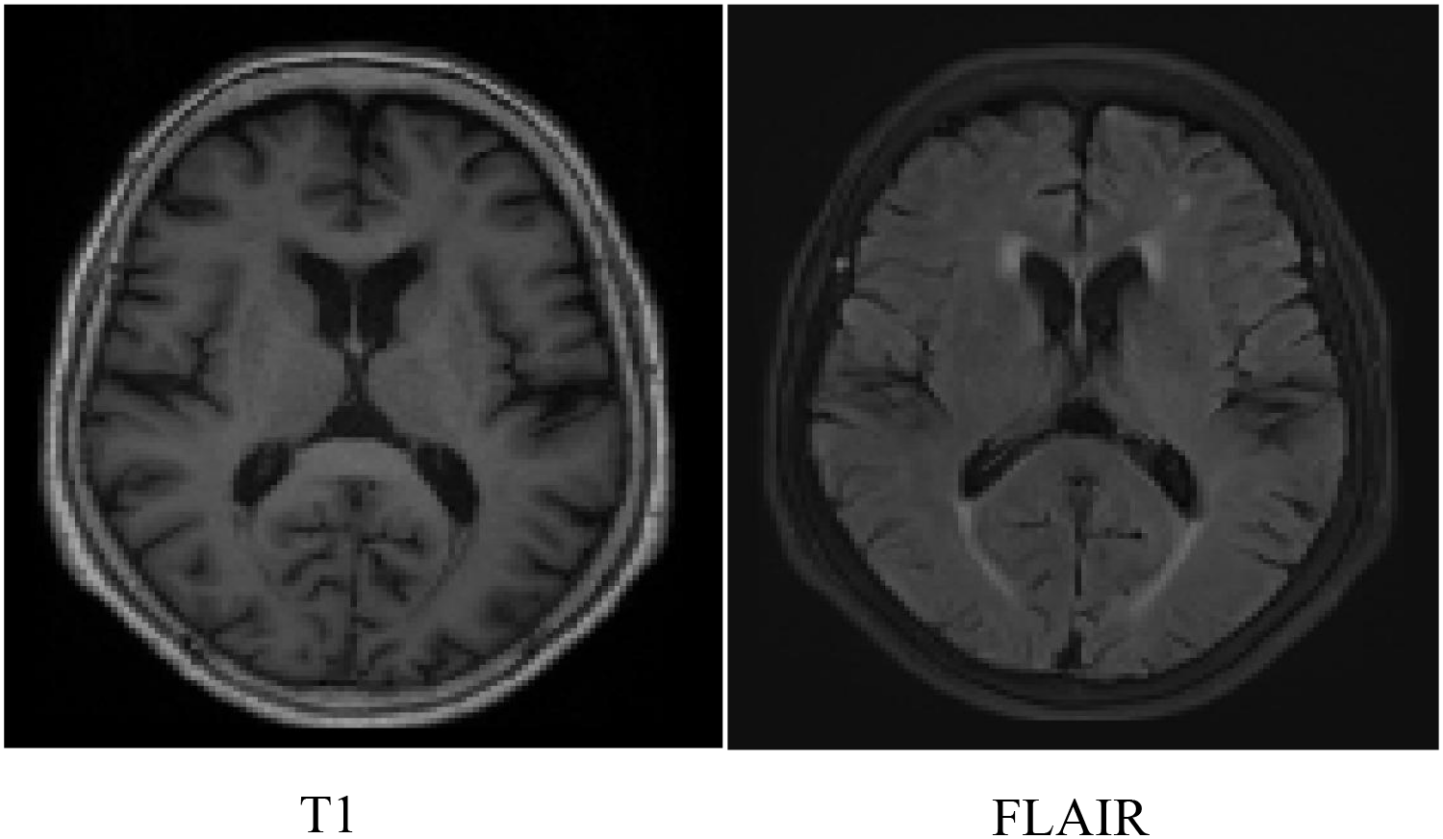
MRI image.

T1-weighted and T2-fluid-attenuated inversion recovery (FLAIR) images were acquired using a Siemens 3.0T MRI scanner, such as **Figure 1**. Imaging parameters were as follows: For T1-weighted imaging, in-plane resolution was 1 mm × 1 mm, slice thickness was 1 mm, with 192 slices; repetition time (TR) = 2400 ms, echo time (TE) = 2.26 ms, inversion time (TI) = 950 ms, flip angle (FA) = 8°, field of view (FOV) = 256 × 256 mm², and matrix size = 256 × 256. For T2-FLAIR imaging, in-plane resolution was 0.86 mm × 0.86 mm, slice thickness was 5 mm, with 22 slices; TR = 9000 ms, TE = 97 ms, TI = 2498 ms, flip angle = 150°, FOV = 230 × 230 mm², and matrix size = 256 × 256.WMH scoring was conducted under strict double-blind conditions: both radiologists were blinded to participants’ genotype status and all clinical assessment data—including neuropsychological, behavioral, and functional measures—to prevent bias in visual rating. Scoring relied exclusively on morphological features of WMH on T2-weighted and FLAIR MRI sequences. Inter-rater reliability was quantified using Cohen’s kappa (κ), calculated from independent ratings of a randomly selected 20% subsample (n = 42). The observed κ = 0.73 (95% CI [0.65, 0.81]) indicates substantial agreement between raters. WMH were evaluated using the Fazekas scale (total range: 0–6 points), which separately assesses Periventricular white matter hyperintensity (PWMH)and deep white matter hyperintensity(DWMH).PWMH grading: 0 – no lesions; 1 – cap-like or pencil-thin lining; 2 – smooth halo-shaped lesions; 3 – irregular periventricular signal extending into deep white matter. DWMH grading: 0 – no lesions; 1 – punctate foci; 2 – beginning confluence of lesions; 3 – large confluent areas. The total score ranges from 0 to 6, with higher scores indicating a wider extent and greater severity of WMH involvement.

### APOE genotyping

For ApoE genotyping, 2 mL of fasting venous blood was collected in the morning from the antecubital vein using EDTA as an anticoagulant. Genomic DNA was extracted using the Whole Blood Genomic DNA Extraction Kit (Shanghai Bioo Technology Co., Ltd., Bioo), followed by targeted amplification of the ApoE gene with the Bioo ApoE Genotyping Kit. Amplified products were mixed with hybridization buffer to prepare the hybridization reaction mixture. Subsequently, antibody solution and chromogenic substrate solution were added sequentially. Hybridization was performed on the BR-526–24 Fully Automatic Nucleic Acid Molecular Hybridization System according to the manufacturer’s protocol. Upon completion, the microarray chip was removed and transferred to a biochip reader. Image acquisition and data analysis were conducted using Bioo’s Arrdy Doctor software for automated interpretation and ApoE genotype assignment.

### Statistical analysis

Data analysis was performed using SPSS 25.0. Normality of continuous variables was assessed using the Shapiro-Wilk test. For variables that significantly deviated from normal distribution, group comparisons were conducted using the Mann-Whitney U test. Categorical variables were analyzed using the chi-square test for independence. Inter-group differences among normally distributed continuous variables were examined using one-way ANOVA with *post hoc* pairwise comparisons as appropriate. To evaluate the mediating role of WMH, a mediation model was constructed using Model 4 of the PROCESS 4.0 macro for SPSS. The indirect effect was estimated via bias-corrected bootstrap resampling with 5,000 samples, and statistical significance was set at p < 0.05.

## Results

A total of 325 right-handed participants were included in this study, with a mean age of 74.85 ± 9.636 years. The mean scores for CMMSE, ADL, and NPI were 14.08 ± 8.058, 39.89 ± 16.886, and 22.8 ± 21.511. After adjusting for age and sex, analyses of covariance (ANCOVA) were conducted to examine the association between APOE gene subtypes and cognitive, functional, and behavioral outcomes as measured by CMMSE, ADL, and NPI total scores. Significant differences across APOE genotype groups were observed for CMMSE (P < 0.05) and ADL (P < 0.05), but not for the total NPI score (P > 0.05) (**Table 1**). With respect to individual neuropsychiatric symptoms (NPS), significant intergroup differences were found in agitation. (P < 0.05)(**Table 2**). Notably, individuals with the ϵ4/ϵ4 genotype exhibited significantly higher levels of delusions, agitation, and irritability compared to those with other ϵ4/ϵ4 subtypes (P < 0.05) (**Table 3**). (**Table 4** is a supplement to **Table 3**).

**Table 1:** Correlation between the scale and APOE genotype.

scale		Sum of squares	Degrees of freedom	Meansquare	F	Significance
CMMSE	Between groups	679.543	4	169.886	2.670	.032 *
	Within the group	20358.377	320	63.620		
	Total	21037.920	324			
ADL	Between groups	3755.260	4	938.815	3.390	.010 *
	Within the group	88628.753	320	276.965		
	Total	92384.012	324			
NPI	Between groups	4176.765	4	1044.191	2.293	.059
	Within groups	145749.832	320	455.468		
	Total	149926.597	324			

*P < 0.05 was statistically significant.

**Table 2:** Correlation between APOE genotype and NPS.

NPS		Sum of Squares	DF	Mean Square	F	Significance	Bonferroni p
Anxiety	Between Groups	266.520	4	66.630	1.171	.324	1.62
	Within Groups	18154.060	319	56.909			
	Total	18420.580	323				
Apathy	Between Groups	49.106	4	12.276	.973	.423	2.115
	Within Groups	4038.587	320	12.621			
	Total	4087.692	324				
Abnormal motor activity	Between Groups	54.774	4	13.693	.711	.585	2.925
	Within Groups	6165.226	320	19.266			
	Total	6220.000	324				
Eating disorders	Between Groups	27.711	4	6.928	1.089	.362	1.8
	Within Groups	2036.277	320	6.363			
	Total	2063.988	324				
Delusion	Between Groups	202.773	4	50.693	2.791	.026*	0.13
	Within Groups	5812.255	320	18.163			
	Total	6015.028	324				
Depression	Between Groups	17.456	4	4.364	.443	.778	3.89
	Within Groups	3152.741	320	9.852			
	Total	3170.197	324				
Nocturnal behavioral disturbances	Between Groups	75.584	4	18.896	1.135	.340	1.7
	Within Groups	5329.339	320	16.654			
	Total	5404.923	324				
Agitation	Between Groups	145.797	4	36.449	4.030	.003*	0.015*
	Within Groups	2894.406	320	9.045			
	Total	3040.203	324				
Irritability	Between Groups	138.160	4	34.540	2.706	.030*	0.15
	Within Groups	4084.929	320	12.765			
	Total	4223.089	324				
Hallucination	Between Groups	123.688	4	30.922	2.278	.061	0.305
	Within Groups	4343.684	320	13.574			
	Total	4467.372	324				
Euphoria	Between Groups	1.094	4	.273	.298	.879	4.395
	Within Groups	293.817	320	.918			
	Total	294.911	324				
Disinhibition	Between Groups	14.827	4	3.707	1.426	.225	1.125
	Within Groups	831.561	320	2.599			
	Total	846.388	324				

*P < 0.05 was statistically significant.

**Table 3:** Differences in NPS symptom manifestations among different APOE gene subtypes.

Dunnett T3								
Dependent variable	APOE genotype	APOE genotype	Mean difference	Standard error	Significance	Bonferroni p	95%confidence interval	
							Lower bound	Upper bound
	23	24	-3.27	2.323	0.796	3.98	-12.21	5.67
		33	-1.257	0.668	0.475	2.375	-3.21	0.69
Delusion		34	-1.283	0.685	0.48	2.4	-3.28	0.71
		44	-3.556*	0.977	.007*	0.035*	-6.42	-0.69
	44	23	2.735*	0.793	.011*	0.055	0.42	5.05
		24	2.703*	0.833	0.039	0.195	0.09	5.31
		33	2.464*	0.634	.004*	0.02*	0.58	4.35
Irritability		34	2.268*	0.65	.012*	0.03*	0.34	4.19
	44	23	2.623*	0.886	.045*	0.225	0.03	5.21
		24	3.093	1.079	0.121	0.605	-0.57	6.76
Agitation		33	2.238*	0.649	.013*	0.065	0.33	4.15
		34	2.345*	0.647	.008*	0.04*	0.44	4.25
	44	23	-0.259	0.224	0.931	4.655	-0.94	0.42
		24	0	0	.*	.*	0	0
Euphoria		33	-0.146	0.094	0.723	3.615	-0.41	0.12
		34	-0.165	0.073	0.218	1.09	-0.37	0.04
Disinhibition	23	24	0	0	.*	.*	0	0
		33	-.389*	0.136	.047*	0.235	-0.77	0
		34	-0.413	0.154	0.08	0.4	-0.85	0.03
		44	-1	0.404	0.173	0.865	-2.23	0.23

*P < 0.05 was statistically significant.

**Table 4:** Descriptive statistics for each genotype group in the main results.

APOE Genotype	quartile	MMSE	ADL	Delusion	Agitation	Irritability	Euphoria	Disinhibition
E2/E3(27)	25	7	29	0	0	0	0	0
	50	18	37	0	0	1	0	0
	75	23	51	4	0	4	0	0
E2/E4(7)	25	11	22	0	0	0	0	0
	50	14	42	0	0	0	0	0
	75	20	49	12	2	2	0	0
E3/E3(144)	25	8.75	27.5	0	0	0	0	0
	50	13	37	0	0	0	0	0
	75	21	51	6	0	4	0	0
E3/E4(121)	25	7.25	23	0	0	0	0	0
	50	14	35.5	0	0	0	0	0
	75	20	52	4	0	4	0	0
E4/E4(26)	25	8.75	24	0	0	0	0	0
	50	13.5	30.5	0	0	3	0	0
	75	20.25	40	0	2.5	6	0	0

(**Table 4** is a supplement to **Table 3**).

To elucidate the mechanisms by which APOE genotypes influence cognition, daily functioning, and neuropsychiatric symptoms, we employed structural equation modeling (SEM) with WMH specified as a candidate mediating variable. Mediation analyses were conducted using Model 4 of the SPSS PROCESS macro (Hayes, 2018), with bias-corrected bootstrapping (5,000 resamples) to estimate 95% confidence intervals (CIs) for indirect effects. In all models, APOE genotype was the independent variable (X), total scores on cognitive (CMMSE) and functional (ADL) scales—or symptom severity scores for delusion, agitation, irritability, and hallucination—served as dependent variables (Y), and WMH volume served as the mediator (M). Age and sex (male = 1, female = 0) were included as covariates. An indirect effect was considered statistically significant if its 95% CI excluded zero. As presented in **Tables 5**–**10**, APOE genotype exhibited robust direct associations with all outcomes; however, none of the indirect pathways via WMH reached statistical significance—indicating that WMH does not mediate the observed relationships between APOE genotype and the clinical measures assessed. .

**Table 5:** Y=CMMSE scale score.

Model effect	Effect	se	t	p	LLCI	ULCI
Total effect	-.3140	.0969	-3.2413	0.0013*	-0.5047	-0.1234
Direct effects	-.2943	.0971	-3.0297	0.0026*	-0.4855	-0.1032
Indirect effects	-.0197	.0153			-0.0539	0.0045

P< 0.05 was statistically significant.

**Table 6:** Y=ADL scale score.

Model effect	Effect	se	t	p	LLCI	ULCI
Total effect	.5598	.1971	2.8397	0.0048*	0.172	0.9477
Direct effects	.5067	.1969	2.5734	0.0105*	0.1193	0.8941
Indirect effects	.0531	.0374			-0.0038	0.1413

P< 0.05 was statistically significant.

**Table 7:** Y=delusion scale score.

Model effect	Effect	se	t	p	LLCI	ULCI
Total effect	.1544	.0517	2.9874	0.003*	0.0527	0.2562
Direct effects	.1436	.0518	2.771	0.0059*	0.0416	0.2455
Indirect effects	.0109	.0094			-0.002	0.0332

P< 0.05 was statistically significant.

**Table 8:** Y=irritability scale score.

Model effect	Effect	se	t	p	LLCI	ULCI
Total effect	.1294	.0371	3.4898	0.0006*	0.0565	0.2024
Direct effects	.1203	.0371	3.2427	0.0013*	0.0473	0.1933
Indirect effects	.0091	.0072			-0.0013	0.0267

P< 0.05 was statistically significant.

**Table 9:** Y=agitation scale score.

Model effect	tbcolw 70ptEffect	se	t	p	LLCI	ULCI
Total effect	.1338	.0438	3.0541	0.0024*	0.0476	0.2199
Direct effects	.1238	.0439	2.8229	0.0051*	0.0375	0.2101
Indirect effects	.0100	.0082			-0.0015	0.0299

P< 0.05 was statistically significant.

**Table 10:** Y=euphoria scale score.

Model effect	tbcolw 70ptEffect	se	t	p	LLCI	ULCI
Total effect	.0516	.0198	2.5997	0.0098*	0.0125	0.0906
Direct effects	.0511	.0200	2.5568	0.011*	0.0118	0.0904
Indirect effects	.0005	.0033			-0.0067	0.0071

P< 0.05 was statistically significant.

## Discussion

Our findings reveal significant group differences in CMMSE and ADL scores between AD patients carrying the APOE ϵ4 allele and non-carriers, suggesting that ϵ4 carriage is associated with greater cognitive impairment and greater functional dependency. These associations align with established evidence from prior literature. Regarding individual NPS, we detected statistically significant differences in agitation severity across APOE genotypic subgroups. Further stratified analyses showed that delusions, agitation, irritability, and euphoria were significantly more severe in ϵ4/ϵ4 homozygotes than in ϵ3/ϵ3, ϵ3/ϵ4, or ϵ2-carrying individuals. Notably, AD patients homozygous for APOE ϵ4 exhibited the highest symptom severity scores across these four domains—indicating a gene-dose–dependent amplification of specific NPS. Collectively, the APOE ϵ4 allele—especially in the homozygous state—appears to confer dual vulnerability: worse cognitive and functional outcomes, alongside heightened risk for a circumscribed profile of NPS, including delusions, agitation, irritability, and euphoria. Crucially, mediation analyses revealed no significant indirect effects of APOE genotype on cognition, daily functioning, or these NPS through WMH, a validated neuroimaging marker of cerebral small vessel disease—thereby supporting direct, WMH-independent pathways.

The neurobiological mechanisms underlying NPS in AD remain poorly understood and inconsistently reported across studies—potentially reflecting the frequent co-occurrence of multiple NPS within individual patients, which contributes to substantial clinical heterogeneity. Moreover, the temporal dissociation between NPS onset and the trajectory of cognitive decline in AD suggests that these symptoms may arise via pathophysiological pathways at least partially distinct from those driving core neurodegenerative pathology. Regarding affective syndromes—including anxiety and depression—while several cross-sectional studies have reported associations between APOE ϵ4 carriage and elevated prevalence or severity of these symptoms in AD (17), such findings lack robust longitudinal support: only two prospective studies to date have examined this relationship, and both failed to detect a significant association (18, 19). Consistent with this more rigorous longitudinal evidence, our analysis revealed no statistically significant association between APOE ϵ4 status (carrier vs. non-carrier) and affective symptom burden in AD patients. Our findings indicate that the APOE ϵ4 allele is significantly associated with increased prevalence and severity of NPS—including delusions, agitation, irritability, and euphoria—in patients with AD. Notably, individuals homozygous for APOE ϵ4 (ϵ4/ϵ4) exhibit markedly more pronounced manifestations of these symptoms compared to heterozygous carriers or non-carriers. Such symptoms represent among the most clinically burdensome and functionally disruptive features of NPS in AD. These results align with prior evidence suggesting that APOE ϵ4 carriers with NPS are at elevated risk for hallucinations and abnormal motor behaviors (11). Nevertheless, few studies have explicitly investigated the differential neuropsychiatric impact of the ϵ4/ϵ4 genotype relative to other APOE genotypes—a gap potentially attributable to the relatively low population prevalence of the ϵ4/ϵ4 variant.

In our cohort of 325 patients with AD, only 26 were homozygous for the APOE ϵ4 allele (ϵ4/ϵ4), underscoring the limited statistical power to detect genotype-specific effects and highlighting the necessity of larger, adequately powered multicenter studies for robust validation. Regarding neuropsychiatric symptomatology, prior evidence consistently associates depression, abnormal motor behavior, anxiety, nocturnal disturbances, and apathy with greater WMH burden (20). Longitudinal mixed-effects modeling revealed a statistically significant association between baseline WMH volume and the rate of increase in total NPI scores over time. Furthermore, higher baseline WMH was independently associated with steeper longitudinal increases in delusions, hallucinations, agitation, depression, and irritability (5). Collectively, these findings emphasize the importance of clarifying the interplay among APOE genotype, WMH burden, and the emergence and progression of NPS in AD. Crucially, formal mediation analysis did not support WMH as a mediator of the APOE ϵ4–NPS relationship; rather, both APOE ϵ4 carrier status and WMH burden emerged as independent, additive predictors of NPS severity.

APOE genotyping holds significant clinical utility, particularly in the context of AD risk stratification and prognostic counseling. The APOE ϵ4 allele is a well-validated genetic predictor of accelerated progression from mild cognitive impairment (MCI) to AD dementia and is independently associated with greater deficits in both cognitive performance and functional independence—specifically in domains of memory, executive function, and activities of daily living. Moreover, ϵ4 carriers with established cognitive impairment consistently demonstrate higher burden and faster progression of neuropsychiatric symptoms, including delusions, agitation, irritability and disinhibition. Collectively, these genotype–phenotype associations underscore the potential value of incorporating APOE status into personalized clinical management frameworks—especially for individuals at elevated genetic risk—to enable earlier symptom monitoring, targeted nonpharmacologic interventions, and more informed shared decision-making.

## Data Availability

The original contributions presented in the study are included in the article/supplementary material. Further inquiries can be directed to the corresponding author.
